# MetaRNN: differentiating rare pathogenic and rare benign missense SNVs and InDels using deep learning

**DOI:** 10.1186/s13073-022-01120-z

**Published:** 2022-10-08

**Authors:** Chang Li, Degui Zhi, Kai Wang, Xiaoming Liu

**Affiliations:** 1 USF Genomics & College of Public Health, University of South Florida, 3720 Spectrum Boulevard, Suite 304, Tampa, FL 33612 USA; 2 School of Biomedical Informatics, The University of Texas Health Science Center at Houston, Houston, TX USA; 3 Children’s Hospital of Philadelphia & Perelman School of Medicine, University of Pennsylvania, Philadelphia, PA USA

**Keywords:** Rare variant, Pathogenicity, Deep learning, Machine learning, Insertion, Deletion, Single nucleotide variant

## Abstract

Multiple computational approaches have been developed to improve our understanding of genetic variants. However, their ability to identify rare pathogenic variants from rare benign ones is still lacking. Using context annotations and deep learning methods, we present pathogenicity prediction models, MetaRNN and MetaRNN-indel, to help identify and prioritize rare nonsynonymous single nucleotide variants (nsSNVs) and non-frameshift insertion/deletions (nfINDELs). We use independent test sets to demonstrate that these new models outperform state-of-the-art competitors and achieve a more interpretable score distribution. Importantly, prediction scores from both models are comparable, enabling easy adoption of integrated genotype-phenotype association analysis methods. All pre-computed nsSNV scores are available at http://www.liulab.science/MetaRNN. The stand-alone program is also available at https://github.com/Chang-Li2019/MetaRNN.

**Supplementary Information:**

The online version contains supplementary material available at 10.1186/s13073-022-01120-z.

## Background

Next-generation sequencing (NGS) has dramatically improved our ability to detect genetic variants in the human genome. However, our current ability to detect genetic variants far exceeds our ability to interpret them, which is one of the significant gaps in effectively utilizing NGS data [1]. This issue is prominent for rare genetic variants (allele frequency <1%) since traditional methods, such as population-based genome-wide association and whole-exome sequencing studies, lack the power to identify rare pathogenic or causal variants from rare benign variants. The issue is especially prominent when the phenotype of interest has low prevalence, such as rare Mendelian disorders where only the proband’s and the parents’ genetic testing data are available. Amino acid changing variants are probably the most well-studied candidate variant type for pathogenic variants. However, as each healthy individual carries approximately 10,000 such variants, hundreds of them are singletons [2], it is still challenging to correctly identify pathogenic causal variants from benign and non-functional ones in the coding regions. Nonsynonymous single nucleotide variants (nsSNVs) and non-frameshift insertion/deletions (nfINDELs) are two types of amino acid changing variants that can exhibit a wide range of functional consequences, from completely neutral and non-functional to protein damaging, which eventually cause severe diseases. This variability makes classifying them in terms of pathogenicity very challenging.

Because experimentally validating the effects of these variants is highly time-consuming and costly, computational approaches have been developed for this purpose [3–18]. These methods can be loosely categorized into three groups: functional prediction methods, which model the functional importance of the variants; conservation-based methods, which use evolutionary data to identify functional regions and variants; and ensemble methods, which combine multiple individual prediction tools into a single more powerful predictor. While these methods have been widely used to predict potentially pathogenic variants, there are still two significant limitations in their application to whole-exome sequencing studies. First, most of these methods either deployed models trained with rare pathogenic and common benign variants or ignored the importance of observed allele frequencies as features, leading to less optimized performance for separating rare pathogenic and rare benign variants. Second, most methods provide prediction scores for only nsSNVs or incomparable scores for nsSNVs and nfINDELs separately, making it infeasible to use these scores as weights in an integrated (nsSNV+nfINDELs) burden test for genotype-phenotype association analysis.

This study developed the MetaRNN and MetaRNN-indel models to overcome these limitations, enabling users to easily annotate and score both nsSNVs and nfINDELs. As predictive features, our classifiers combine recently developed independent prediction algorithms, conservation scores, and allele frequency information from the 1000 Genomes Project (1000GP) [19], ExAC [20], and gnomAD [21]. Annotations from flanking ± 1 codon of nucleotides around the target variants were extracted by bidirectional gated recurrent units [22] (GRUs). We trained our recurrent neural network (RNN) model with 26,517 nsSNVs (absent from at least one of the three population datasets, namely gnomAD, ExAC, and 1000GP) and 1981 nfINDELs reported in ClinVar [23] on or before 20190102. To evaluate the performance of the proposed models, we compared multiple state-of-the-art computational methods using independent test sets constructed from well-known variation-disease association databases, i.e., ClinVar [23] and HGMD [24], a TP53 functional mutation dataset [25], and a dataset of potential cancer driver variants [26]. Our results suggest that utilizing flanking region annotations helps boost model performance for separating rare pathogenic variants versus rare (and common) benign variants. In addition, we provide pre-computed MetaRNN scores for all possible human nsSNVs available at https://sites.google.com/site/jpopgen/dbNSFP [27, 28]. A GitHub page for a stand-alone annotation software package for both nsSNVs and nfINDELs is available at https://github.com/Chang-Li2019/MetaRNN [29].

## Methods

### Training sets

ClinVar database files clinvar_20190102.vcf.gz and clinvar_20200609.vcf.gz were downloaded from https://www.ncbi.nlm.nih.gov/clinvar/ [23] under the GRCh38/hg38 genome assembly. Variants in the older file were used in the training phase of model development. Next, we prepared separate training sets for point variants and insertion/deletions (InDels). For SNVs, nonsynonymous SNVs (nsSNVs) labeled “Pathogenic” or “Likely pathogenic” were used as true positives (TPs), and nsSNVs labeled “Benign” or “Likely benign” were used as true negatives (TNs). Variants with conflicting clinical interpretations were removed. Conflicting clinical interpretations were defined as one of these scenarios: conflict between benign/likely benign and variants of unknown significance (VUS), conflict between pathogenic/likely pathogenic and VUS, or conflict between benign/likely benign and pathogenic/likely pathogenic. Variants that were absent from at least one of the three datasets (gnomAD [21], ExAC [20], and the 1000 Genomes Project [19]) were retained. A further filter removed any nsSNVs that were absent in all three datasets. We consider this to be a good trade-off between preserving important allele frequency information and removing “easy-to-classify” variants during training. In the end, 26,517 rare nsSNVs with 9009 TPs and 17,508 TNs (Additional file 1: Table S1) were used for training. For InDels, the same criteria were applied to obtain TPs and TNs. Additionally, only InDels annotated as non-frameshift (nfINDELs) and having lengths >1 and ≤ 48 base pairs were included. A total of 1981 rare nfINDELs with 1306 TPs and 675 TNs passed the filtering criteria and were used to train the MetaRNN-indel model (https://github.com/Chang-Li2019/MetaRNN) [29] (Additional file 1: Table S2).

### Test sets

We constructed 7 test sets to evaluate the performance of our SNV-based model, namely, MetaRNN (https://github.com/Chang-Li2019/MetaRNN) [29] (summary in Additional file 1: Table S3) with 24 other methods, including MutationTaster [10], FATHMM [30], FATHMM-XF [12], VEST4 [9], MetaSVM [31], MetaLR [31], M-CAP [17], REVEL [4], MutPred [16], MVP [8], PrimateAI [15], DEOGEN2 [14], BayesDel_addAF [7], ClinPred [6], LIST-S2 [5], CADD [3], Eigen [13], GERP [32], phyloP100way_vertebrate [33], phyloP30way_mammalian, phyloP17way_primate, phastCons100way_vertebrate, phastCons30way_mammalian, and phastCons17way_primate. The first test set (rare nsSNV test set, RNTS) was constructed from rare pathogenic nsSNVs with a maximum population allele frequency (AF) of 0.01 that were added to the ClinVar database after 20190102 and rare nsSNVs with a maximum population AF of 0.01 that were present in all three population datasets while not reported in ClinVar and matching on genomic location (randomly selected non-pathogenic nsSNVs within 10 kb from the pathogenic ones), resulting in 11,540 variants with 5770 TPs and 5770 TNs (Additional file 1: Table S4). The second test set (rare clinvar-only test set, RCTS) was constructed from recently curated (after 20190102) ClinVar rare pathogenic nsSNVs (*n* = 6190) and rare benign nsSNVs defined as having a maximum AF<0.01 in all population datasets (*n* = 11,811) (Additional file 1: Table S5). The third test set (de novo RCTS, DN-RCTS) was constructed from RCTS with 0 AF in all population datasets, which resulted in 4537 TPs and 831 TNs (Additional file 1: Table S6). The fourth test set (all allele frequency set, AAFS) was constructed from all pathogenic and benign nsSNVs added to the ClinVar database after 20190102 regardless of AF, resulting in 29,924 variants with 6205 TPs and 22,808 TNs (Additional file 1: Table S7). The fifth test set, the TP53 test set (TP53TS), was constructed from the TP53 mutation website (https://p53.fr/index.php) [34]. Variants with median activity <50 were considered pathogenic, while variants with median activity ≥100 were considered benign. After removing variants used in the training set, 824 variants remained with 385 TPs and 439 TNs (Additional file 1: Table S8). The sixth test set was retrieved from a recent publication (Additional file 1: Table S9) [26]. The TPs (*n* = 878) were curated from cancer somatic variant hotspots, and the TNs (*n* = 1756) were curated from the population sequencing study DiscovEHR [35]. The Human Gene Mutation Database (HGMD) (https://www.hgmd.cf.ac.uk/) [24] is another popular database that provides high-quality disease-associated variants. As the last test set, we retrieved all DM variants (disease mutation; the class of variants in HGMD with the highest confidence of being pathogenic) from HGMD Professional version 2021.01. Only variants reported in dbNSFP (https://sites.google.com/site/jpopgen/dbNSFP) [27, 28] as missense were kept. We further removed variants that were reported in the HGMD Professional version 2017 to avoid unfair comparisons with scores that used HGMD in their training process. Additionally, variants reported in ClinVar 20200609 as pathogenic, likely pathogenic, benign, or likely benign were filtered out to explore the generalizability of our score to independently curated disease-causing variants. These filtered nsSNVs were used as TPs. For true negatives (TNs), we used rare nsSNVs that were observed in gnomAD v3 with allele frequencies between 0.01 and 0.0001 as a trade-off between their rarity and probability of being truly benign. The number of TP and TN variants were matched using random selection, which resulted in 45,256 nsSNVs in total (22,628 TP variants and 22,628 TN variants). For our InDel-based model, namely, MetaRNN-indel, the first test set was constructed from InDels added to the ClinVar database after 20190102, which resulted in 828 InDels with 365 TPs and 463 TNs (Additional file 1: Table S10). The second test set was constructed from HGMD Professional version 2021.01. All the nfINDELs that were not used in training MetaRNN-indel were kept as TP. For TN, rare nfINDELs with AF less than 0.01 were retrieved from gnomAD v2.1.1 as TNs, which were then randomly sampled to match the number of TPs. A total of 8020 nfINDELs (4010 TP variants and 4010 TN variants) were collected after filtering.

### Flanking nsSNVs

**Fig. 1:**
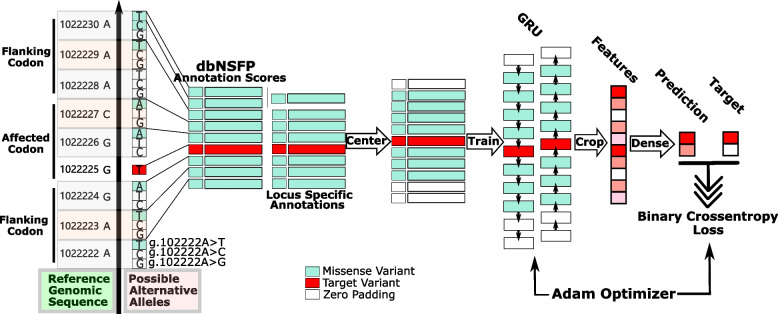
Data preparation and model development steps for MetaRNN. First, the target variant and affect codon were identified, e.g., g.1022225G>T. Second, the flanking sequences were retrieved as well as all possible alternative alleles, as illustrated on the nucleotides to the right of the up-pointing arrow. Third, only alleles that result in a missense variant were kept, and annotation scores were averaged across these alleles within the same locus. For example, the annotation scores for variants g.1022230A>T and g.1022230A>C will be averaged, to get the locus-specific annotations. Lastly, the model will be trained using the annotations for context variants and annotations for the target variant

After obtaining all data sets of target variants, we retrieved nsSNVs from their flanking sequences using dbNSFP4.1a [27, 28]. Specifically, the genomic location of the variant and the affected amino acid position of the protein and the affected codon were first identified in dbNSFP. Then, a window size of ± 1 codon around the affected codon was identified, and all nsSNVs inside this window were retrieved with a maximum length of 9 base pairs (bps). For a given target variant, the maximum possible number of nucleotides on either side is 5 bps (3 bps from one flanking codon and 2 bps from the target codon). To center the input window on the target variant and have a uniform shape for all inputs, we padded the input window to reach an 11-bp window for each target variant so that there were 5 bps on each side of the target variant. This window, including the target variants, was used as one input for our model (Fig. 1).

For each position, multiple alternative alleles may exist. For each annotation at context loci, we calculated the average score across all alleles that would result in a nonsynonymous variant at the locus. This averaged annotation score was then used to represent the locus for that annotation. This setup has the advantage of keeping the most critical context information while limiting the unnecessary noise introduced by having inconsistent order and dimension of alleles at different loci, e.g., some loci may possess three nsSNVs while others may include only one nsSNV. The target variant would directly use annotation scores for the observed allele. We assume that these nsSNVs and related annotations can capture the most critical context information concerning the pathogenicity and functional importance of the amino acids. We assumed that context nsSNVs provided all the critical information, so we ignored any synonymous variants in composing the context information. After these steps, the input dimension for the MetaRNN model becomes 11 (bps) by 28 (features, see below).

The same rules to adopt the flanking region were applied to InDels with one difference: instead of affecting only one codon, target InDels may directly affect multiple codons simultaneously. Thus, the ± 1 codon window was defined as the window beyond all the directly affected codons. For deletions, target variants were those loci deleted by the variant, and their annotations were averaged for each locus. For insertions, since no annotation is available for the inserted nucleotides, we used annotations from loci adjacent to the insertion position as surrogates. Since we focus on short InDels with length ≤ 48, with 5 bps around each side as context information, the input dimension for the MetaRNN-indel model is 58 (bps) by 28 (features, see below). Again, the synonymous variants were ignored in composing the context information.

### Feature selection

For each variant, including target nsSNV/nfINDEL and flanking region nsSNVs, 28 features were either calculated or retrieved from the dbNSFP database, including 16 functional prediction scores: SIFT [36], Polyphen2_HDIV [37], Polyphen2_HVAR, MutationAssessor [11], PROVEAN [38], VEST4 [9], M-CAP [17], REVEL [4], MutPred [16], MVP [8], PrimateAI [15], DEOGEN2 [14], CADD [3], fathmm-XF [12], Eigen [13], and GenoCanyon [39]; eight conservation scores including GERP [40], phyloP100way_vertebrate [33], phyloP30way_mammalian, phyloP17way_primate, phastCons100way_vertebrate, phastCons30way_mammalian, phastCons17way_primate, and SiPhy [41]; and four calculated allele frequency (AF)-related scores. The highest AF values across subpopulations of the four data sets from three studies, namely, the 1000 Genomes Project (1000GP), ExAC, gnomAD exomes, and gnomAD genomes, were used as the AF scores. All missing scores in the dbNSFP database were first imputed using BPCAfill (http://ishiilab.jp/member/oba/tools/BPCAFill.html) [42], and all scores were standardized before feeding to the model for training. Some more recently developed scores were excluded to minimize type I circularity in training our ensemble model, including MPC and ClinPred, which used ClinVar variants in their training process.

### Model development

We applied a recurrent neural network with gated recurrent units [22] (GRU) to extract and learn the context information around target variants (Fig. 1). Bayesian Hyperparameter Optimization [43] was used to determine the best-performing model structure from a wide range of model structures. Specifically, the input layer takes an 11 × 28 matrix as input for the MetaRNN and a 58 × 28 matrix for the MetaRNN-indel model. After the bidirectional GRU layer, the MetaRNN model cropped out the context information, and only the learned features for the target variant were kept. This setup can significantly reduce the number of parameters compared to keeping all context features in the subsequent dense layer. Following the same idea, for MetaRNN-indel, the output for the last bidirectional GRU layer only returns the prediction for the final locus (*return_sequences* parameter was set to false) to limit the number of possible parameters in the following dense layer. The output layer is composed of 1 neuron with a sigmoid activation to model our binary classification problem. A binary cross-entropy loss was used as the loss function, and the Adam optimizer [44] was used to update model parameters through backpropagation [45]. This process used 70% of the training data for model training and 30% of the training data for performance evaluation, so no test sets were used in this step. The Python packages sci-kit-learn (https://scikit-learn.org/stable/) [46] and TensorFlow 2.0 (https://www.TensorFlow.org/) [47] were used to develop the models, and KerasTuner (https://keras-team.github.io/keras-tuner/) [48] was adopted to apply Bayesian Hyperparameter Optimization. The search space for all the hyperparameters is shown in Additional file 1: Table S11. The models with the smallest validation log loss were used as our final models for nsSNV (MetaRNN) and nfINDEL (MetaRNN-indel).

### Comparison of the performance of individual predictors

As a model diagnosis step, SHAP (SHapley Additive exPlanations) values were calculated to measure each feature’s contribution to the predicted consequence of variants [49]. We first used 100 random samples from our training data to calculate the background distribution of the values. Next, feature permutations were performed using 100 random samples from our validation data (RNTS). Since the variance of the estimates scale by 1/sqrt(background sample size), we chose to use 100 samples, which would give a reasonable estimate. The Python library SHAP (https://shap.readthedocs.io/en/latest/index.html)  [49] was used to calculate SHAP values and plot visualizations.

To quantitatively evaluate model performance, we retrieved 39 prediction scores from dbNSFP to compare with our MetaRNN model including MutationTaster [10], MutationAssessor [11], FATHMM [30], FATHMM-MKL [50], FATHMM-XF [12], PROVEAN [38], VEST4 [9], MetaSVM [31], MetaLR [31], M-CAP [17], MPC [18], REVEL [4], MutPred [16], MVP [8], PrimateAI [15], DEOGEN2 [14], BayesDel (AF and noAF models) [7], ClinPred [6], LIST-S2 [5], LRT [51], CADD (raw and hg19 models) [3], DANN [52], Eigen (raw and PC models) [13], GERP [32], Polyphen2 (HVAR and HDIV) [53], SIFT4G [54], SiPhy [41], GenoCanyon [55], fitCons (integrated) [56], phyloP (100way_vertebrate, 30way_mammalian and 17way_primate) [33], and phastCons (100way_vertebrate, 30way_mammalian and 17way_primate) [57]. The corresponding rank scores were retrieved for each of these 39 annotation scores to facilitate comparison. For the MetaRNN-indel model, four popular methods were compared, including DDIG-in (http://sparks-lab.org/server/ddig/) [58], CADD (https://cadd.gs.washington.edu/) [3], PROVEAN (https://www.jcvi.org/research/provean) [38], and VEST4 (http://cravat.us/CRAVAT/) [59]. For the ClinVar holdout test set, all four methods were compared with MetaRNN-indel. For the HGMD test set, VEST4 was removed from the comparison since it used HGMD InDels during training, and we did not have access to an older version of HGMD with InDels to exclude these variants. For both test data sets, LiftOver was used to convert hg38 genomic coordinates to GRCh37/hg19 for DDIG-in and PROVEAN. DDIG-in scores were retrieved from https://sparks-lab.org/server/ddig/ [58]. VEST4 indel scores were retrieved from http://cravat.us/CRAVAT/ [59]. The CRAVAT format was used, and each InDel variant was assumed to be located on both + and – strands. For PROVEAN indel, the scores were retrieved from http://provean.jcvi.org/genome_submit_2.php?species=human [38]. The CADD v1.6 scores under the GRCh38 assembly were obtained from https://cadd.gs.washington.edu/score [3]. We plotted receiver operating characteristic (ROC) curves and calculated the area under the ROC curve (AUC) for each method being compared using our test sets. Additionally, average precision, which summarizes a precision-recall curve, was used to measure test sets with an imbalanced number of TPs and TNs. The Python package matplotlib (https://matplotlib.org/) [60] was used to plot ROC curves, and the Python package sci-kit-learn (https://scikit-learn.org/stable/) [46] was used to calculate AUC scores.

### Development of MetaRNN and MetaRNN-indel stand-alone program

To facilitate custom annotations with user-provided VCF files, we created a GitHub page (https://github.com/Chang-Li2019/MetaRNN) [29] with instructions to run annotations as a stand-alone program. Briefly, the program includes the following steps to make final predictions of MetaRNN and MetaRNN-indel scores. First, it takes as input a VCF file that includes candidate variants. Second, ANNOVAR (https://annovar.openbioinformatics.org/en/latest/) [61] was used to annotate these variants, and only nsSNVs and nfINDELs were retained. Third, for nsSNVs, the program will extract MetaRNN predictions from our database of all pre-calculated nsSNVs; for nfINDELs, the target variant and its context variants will be first identified, and all required annotations will be retrieved from dbNSFP [27], and the MetaRNN-indel model will be used to make predictions on these user-provided nfINDELs. Lastly, an output file will be generated for nsSNVs and nfINDELs separately.

## Results

### Allele frequencies as crucial features in separating pathogenic variants

**Fig. 2:**
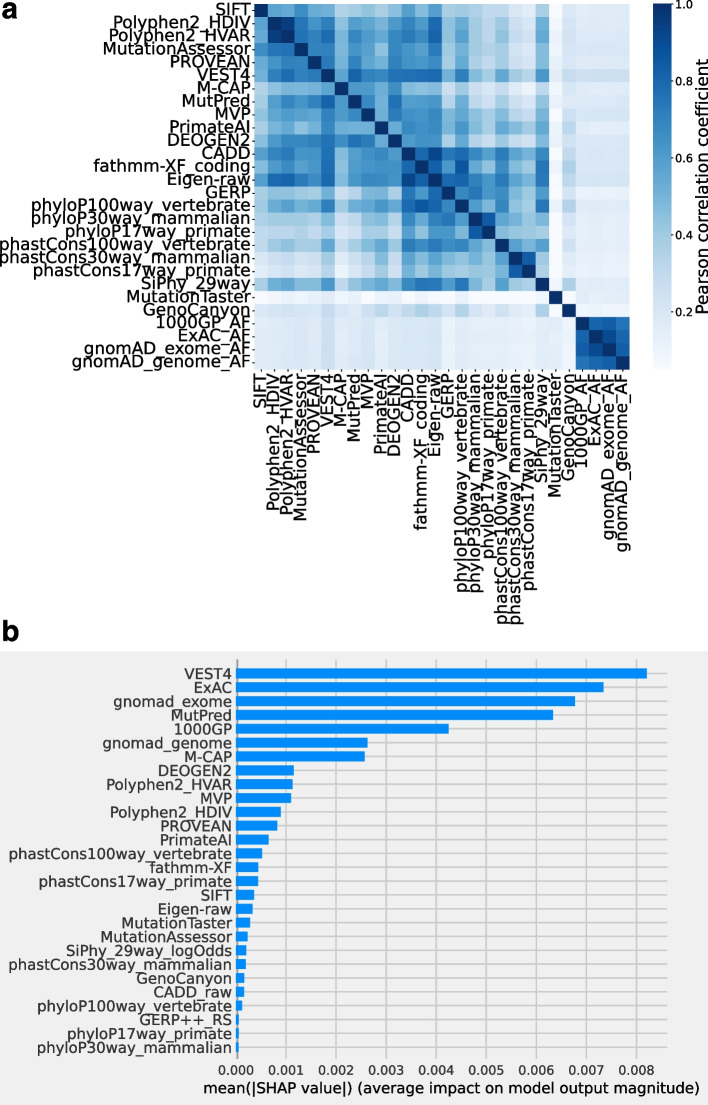
Features used to train MetaRNN and MetaRNN-indel. **a** Correlation between features used to train MetaRNN. **b** Feature importance for all features used in the MetaRNN model

The MetaRNN and MetaRNN-indel models used an ensemble method that combined 24 individual prediction scores and four allele frequency (AF) features from the 1000 Genomes Project (1000GP) [19], ExAC [20], and gnomAD [21]. As shown in Fig. 2a, most of the component conservation scores and ensemble scores showed moderate to strong correlations (correlation coefficient between 0.4 and 1). However, MutationTaster [10] and GenoCanyon [39] showed a weak correlation with all other features. Since most SNVs are not observed in multiple populations (AF=0), correlations between different AF features are also strong (>0.8). AF features showed a weak correlation with all other individual predictors, implying that previous annotation scores have not fully exploited such allele frequency information. This observation is also supported by the feature importance analysis (Fig. 2b). The most important feature is the VEST4 score, which was trained on rare pathogenic variants and common benign variants. The ExAC and gnomAD exome AFs were ranked as the second and third most important features, while AF information from the 1000 GP and gnomAD whole-genome sequencing studies were ranked fifth and sixth, respectively. This observation is in concordance with previous observations [6], highlighting the importance of population AF data in inferring the functional significance of nsSNVs. With the increasing availability of population-based studies, these new AF-based features can complement earlier developed functional annotation tools, such as VEST4.

### Performance comparison of MetaRNN to other predictive algorithms using ClinVar

**Fig. 3:**
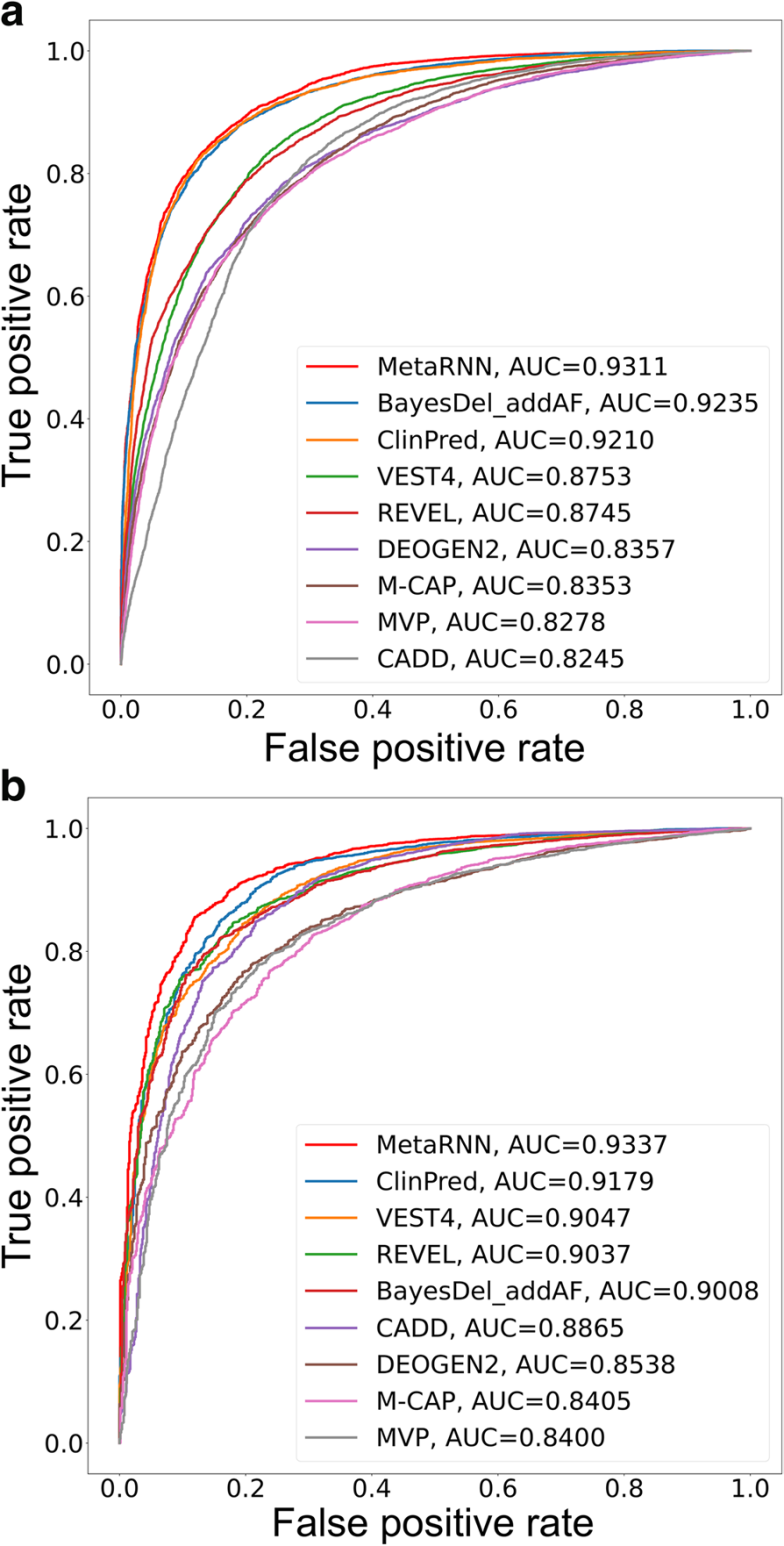
Comparisons of MetaRNN with other prediction tools. **a** Performance comparison of MetaRNN and 8 other nsSNV prediction tools using the rare nsSNV test set (RNTS). **b** Performance comparison of MetaRNN and 8 other nsSNV prediction tools using the de novo ClinVar test set (DN-RCTS)

As the major goal of the MetaRNN model is to separate rare pathogenic from rare benign nsSNVs, we constructed a rare nsSNV test set (RNTS; see the “Methods” section) that was composed of rare (AF<0.01) pathogenic ClinVar nsSNVs after release 20190102 and location-matched rare (AF<0.01) benign nsSNVs from gnomAD, ExAC, and 1000GP. The RNTS (*n* = 11,540) was constructed to simulate the challenge faced by real-world whole-exome sequencing studies where it is crucial to correctly identify potentially pathogenic variants from neutral background variants that both have low AF frequencies in population datasets. For the RNTS set, MetaRNN achieved the best performance with an area under the ROC curve (AUC) equal to 0.9311 in separating these rare nsSNVs, followed by BayesDel_addAF [7] and ClinPred [6] (selected comparisons with eight tools are available in Fig. 3a; all comparisons with 24 tools are available in Additional file 2: Fig. S1). It has been reported that computational tools tend to overestimate the number of pathogenic variants (i.e., high sensitivity and low specificity) [62, 63]. Consequently, we then examined the models’ specificity at 95% sensitivity. The MetaRNN model achieved the best specificity (0.6877) at 95% sensitivity, followed by ClinPred (0.6430) and BayesDel (0.6404).

To comprehensively evaluate the performance of MetaRNN in separating ClinVar reported pathogenic and benign nsSNVs, we constructed 3 test sets for 3 different use scenarios. First, we constructed a de novo rare ClinVar test set (DN-RCTS) where all variants had AF equal to 0 to evaluate MetaRNN’s performance for extremely rare variants or those without available population AF data. As shown in Fig. 3b (selected comparisons with eight tools; all comparisons with 24 tools are available in Additional file 2: Fig. S2), MetaRNN outperformed all competitors with an AUC equal to 0.9337, followed by ClinPred (AUC=0.9179) and VEST4 (AUC=0.9047). We also evaluated the models’ specificity at 95% sensitivity. The MetaRNN model achieved the best specificity (0.6919) at 95% sensitivity, followed by ClinPred (0.6760) and VEST4 (0.5974). As this test set was imbalanced (4537 TPs vs. 831 TNs), a precision-recall curve was plotted, and similar results were observed (Additional file 2: Fig. S3). Second, we constructed a rare ClinVar test set (RCTS) where all variants had AF<0.01 to evaluate MetaRNN’s performance for separating rare variants reported in ClinVar. As shown in Additional file 2: Fig. S4, the MetaRNN performed the best in terms of average precision (AP) and AUC, and ClinPred and BayesDel were not far behind. Lastly, to examine our model’s performance in ClinVar regardless of AF, we constructed an all-allele-frequency set (AAFS) comprised of all available ClinVar pathogenic SNVs and benign SNVs (rare+common) that are not used for model development. As shown in Additional file 2: Fig. S5, using AAFS as the benchmark test set, MetaRNN outperforms all competitors with an AUC of 0.9862. The second-best model was ClinPred (AUC=0.9841), followed by BayesDel (AUC=0.9759). In general, in our ClinVar-based comparisons, ensemble methods and functional predictors outperform conservation-based methods. In addition, MetaRNN showed improved performance under all benchmark settings regardless of the different AF filters used for the inclusion of nsSNVs.

### Investigating the generalizability of MetaRNN to different disease and functional databases

To explore the generalizability of our model to disease-causing nsSNVs curated with different standards, we retrieved disease-causing mutations (DMs) from HGMD Professional v.2021.01 [24] as TPs (*n* = 22,628) and rare nsSNVs observed in gnomAD v3 with allele frequencies between 0.01 and 0.0001 as TNs (*n* = 22,628), which matched the number of TPs. Only variants reported in dbNSFP [27, 28] as missense were kept. To minimize the type I circularity of the data, we further removed variants that were reported from an older version HGMD Professional database (v. 2017). Additionally, variants reported in ClinVar 20200609 as “pathogenic,” “likely pathogenic,” “benign,” or “likely benign” were filtered out. MetaRNN still outperformed other competitors using this test set with an AUC of 0.9689 (Additional file 2: Fig. S6). TP53 is one of the most well-studied human genes, and its functional impact is linked to tumor suppression [34]. Using results from a TP53 mutagenesis assay (*n* = 824), we showed that MetaRNN provides the best estimations for results from such functional experiments with an AUC of 0.8074 (Additional file 2: Fig. S7). Additionally, we collected a test set of cancer somatic variants from a recent study [26] and showed that both MetaRNN and BayesDel showed the best performance in separating potential driver variants from potentially benign variants observed in populations (Additional file 2: Fig. S8) [26]. These results highlighted MetaRNN’s increased ability relative to those of the other methods to separate not only rare pathogenic variants from rare benign ones but also variants with various degrees of functional importance across different disease pathways.

### MetaRNN-indel outperformed competitors in identifying pathogenic nfINDELs

**Fig. 4:**
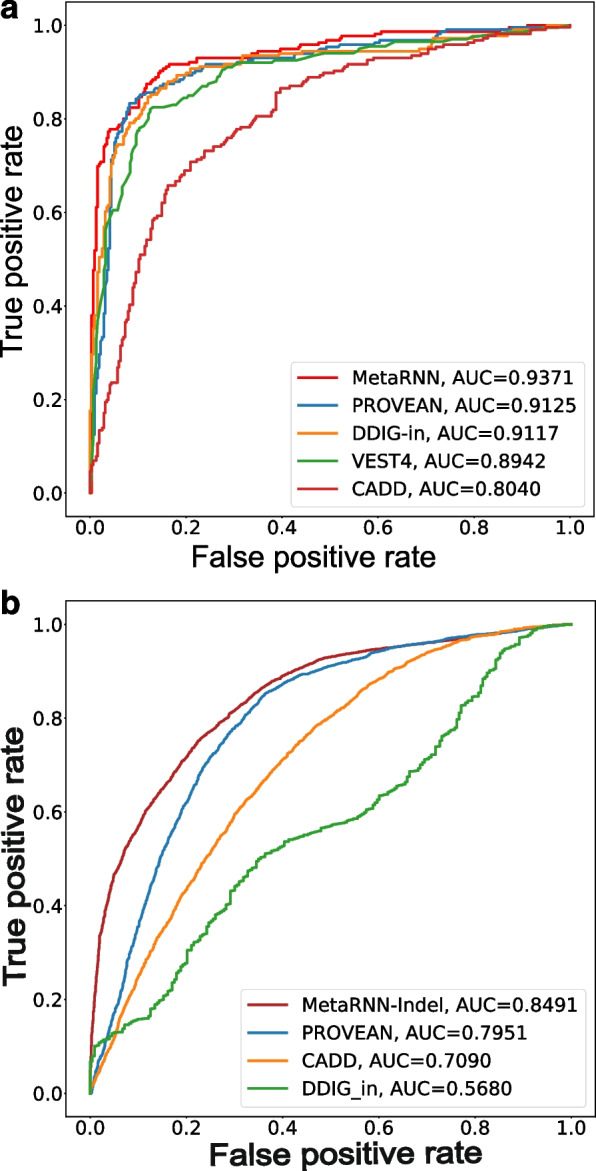
Comparisons of MetaRNN-indel with other prediction tools. **a** Performance comparison of MetaRNN-indel and 4 other nfINDEL prediction tools using the ClinVar test set for nfINDELs. **b** Performance comparison of MetaRNN-indel and other nfINDEL prediction tools using the HGMD test set for nfINDELs. Note that VEST4 was removed in this comparison because it used HGMD during its training process

To examine the performance of MetaRNN-indel, we first curated a test set that was composed of pathogenic ClinVar nfINDELs after release 20190102 (*n* = 828). MetaRNN-indel outperformed all competitors in ranking nfINDELs with an AUC equal to 0.9371 (Fig. 4a), including two methods, VEST [59] and CADD [3], which showed good performance in nsSNV-based analyses. The second test set was constructed from HGMD Professional version 2021.01. All the nfINDELs that were not in the training set of MetaRNN-indel were used as the pathogenic set. For the benign set, rare nfINDELs with AF less than 0.01 were retrieved from gnomAD v2.1.1 and then matched to the number of pathogenic variants. A total of 8020 nfINDELs (4010 pathogenic variants and 4010 benign variants) were collected after filtering. MetaRNN-indel still outperformed other scores with an AUC of 0.8491, followed by PROVEAN (AUC=0.7951) (Fig. 4b).

### MetaRNN showed improved interpretability of variants of unknown significance

**Fig. 5:**
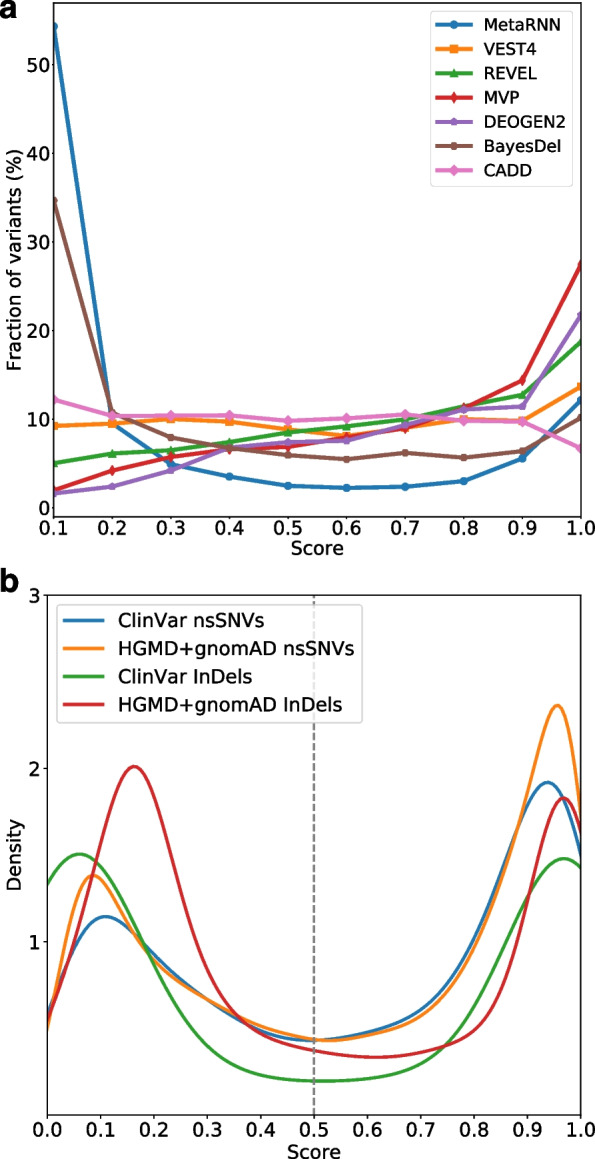
MetaRNN and MetaRNN-indel score distributions in test data sets. **a** Score distribution for ClinVar variants of unknown significance (VUS). **b** Score distributions of MetaRNN and MetaRNN-indel predictions on matched test sets where the number of pathogenic and benign variants are the same

To explore the interpretability and usability of the proposed models, we first predicted scores for all nsSNVs in ClinVar that showed conflicting clinical interpretations (*n* = 20,337). These nsSNVs represent an essential class of variants with unknown significance (VUS) according to the ACMG-AMP guidelines [64]. The ability to distinguish and interpret VUS variants is crucial to the clinical application of the proposed score. A score that shows sufficient dispersion enables further identification of relevant candidate variants. Additionally, these conflicting VUS variants are of interest with some evidence of being either pathogenic or benign. Among these variants, 15,788 (77.6%) showed conflicting interpretations between benign/likely benign and unknown significance (“benign conflicting group”), whereas 4110 (20.2%) showed conflicting interpretations between pathogenic/likely pathogenic and unknown significance (“conflicting pathogenic group”). Based on the fact that the benign conflicting group had approximately four times more variants than the conflicting pathogenic group, we expect that variant prediction tools should reflect this observation. While other scores either showed little change in the distribution across their predictions (e.g., CADD [3], VEST [9], REVEL [4]) or potentially underestimated the proportion of VUSs at the extremes (BayesDel [7]), MetaRNN’s predictions showed a score distribution that fit these assumptions (Fig. 5a), which peaked at the extremes of its score range and had approximately four times more extreme benign predictions than extreme pathogenic predictions.

### Compatibility of MetaRNN and MetaRNN-indel scores

Additionally, we explored the score distributions for nsSNVs and nfINDELs used in testing the respective MetaRNN models. A clear bimodal distribution was observed for both MetaRNN and MetaRNN-indel predictions (Fig. 5b). Based on a cutoff value of 0.5 as inherited by the sigmoid activation function used in the MetaRNN models, pathogenic nsSNVs and nfINDELs can be effectively separated from benign ones. To further check for compatibility of predictions from both models, defined as the trend that similar prediction scores convey a similar likelihood of being pathogenic, we constructed a combined dataset with 828 randomly sampled prediction scores from the RNTS by MetaRNN and 828 prediction scores from the ClinVar test set for nfINDELs by MetaRNN-indel. We hypothesize that if these two scores are compatible, the AUC calculated using the combined data will perform similarly to the individual AUCs. As shown in Additional file 2: Fig. S9, the combined predictions had an AUC equal to 0.9379, similar to those observed using predictions from individual models (MetaRNN AUC=0.9322, MetaRNN-indel AUC=0.9378). These observations have two implications. First, using a cutoff of 0.5 is in accordance with the interpretation of the scores as probabilities, where a score greater than 0.5 can be categorized as having a higher probability of being pathogenic and a score less than 0.5 can be categorized as having a higher probability of being benign. Second, with a shared cutoff value and similar distributions for nsSNV and nfINDEL scores across independent test sets, we show that predictions from our two models, namely, MetaRNN and MetaRNN-indel, are comparable. This feature can effectively help increase the power of genotype-phenotype association studies and related gene-set association analyses. It can also help fine-mapping the exact causal variants in coding sequences.

### Sensitivity analysis shows MetaRNN’s superior performance over other model structures

**Fig. 6:**
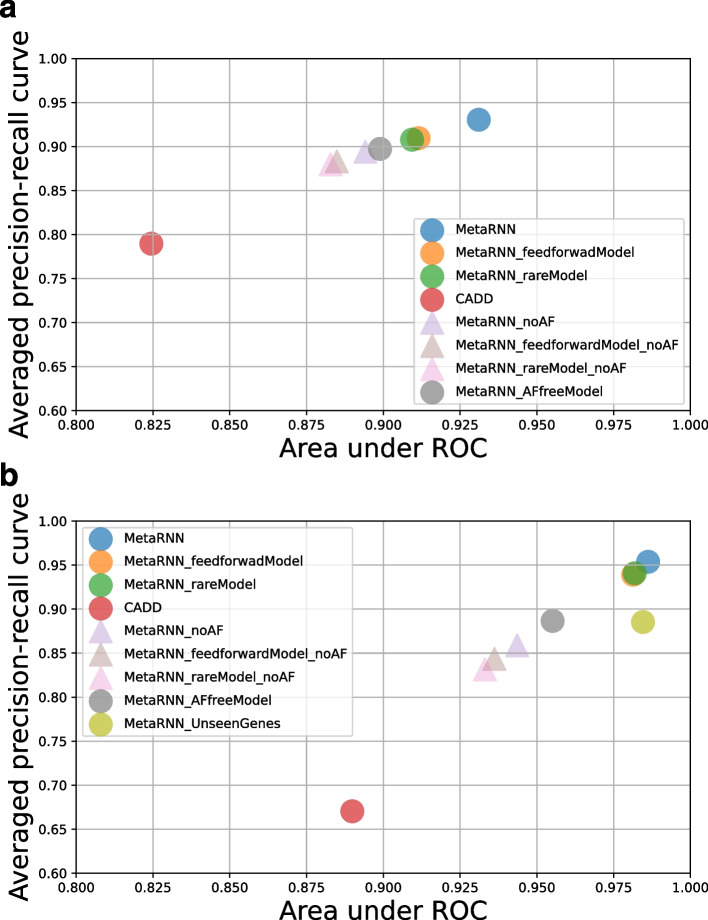
Performance comparison of alternative model setups. **a** RNTS as the benchmark, which has 5770 TPs and 5770 TNs. **b** AAFS as the benchmark, which has 6208 TPs and 22,808 TNs. Triangular shapes indicate *_noAF* models

Finally, to further explore the robustness of our MetaRNN model, we trained multiple alternative models using different setups and tested their performances under various perturbation conditions. First, we trained a model using only rare TPs and TNs with AF<0.01, which included 8937 TPs and 9133 TNs from ClinVar 20190102 (MetaRNN_rareModel). Additionally, an AF-free model was trained, which removed all AF information during training (MetaRNN_AFfreeModel). Both models were trained using the same search spaces as the original MetaRNN model. These two models were used to examine whether a more stringent AF filtering process or dropping AF information completely can improve the model’s performance. Finally, to investigate whether our MetaRNN model, which adopted flanking sequence information and a bidirectional GRU layer, can provide additional predictive power, we trained a feed-forward neural network using only annotations of the target variant (MetaRNN_feedforwardModel). We first evaluated these models using the RNTS test set regarding their average precision-recall (AP) and AUC (Fig. 6a). We found that MetaRNN showed the best performance across both metrics compared with all other model setups. Limiting the training data to only rare variants (MetaRNN_rareModel) and ignoring context information (MetaRNN_feedforwardModel) negatively impacted model performance. For de novo variants, it is expected that AF information from population-based studies is not available. Therefore, we created a perturbation condition that masked all AF information during model evaluation (*_noAF*). As expected, all models’ performances dropped when AF information was removed. For example, MetaRNN’s AUC decreased from ~0.93 to ~0.895. However, even without AF information, MetaRNN can still perform well in separating rare pathogenic and rare benign variants. Moreover, we examined these same conditions using AAFS as a benchmark (Fig. 6b). As shown in the figure, our MetaRNN again performed the best, followed by MetaRNN_feedforwardModel and MetaRNN_rareModel. MetaRNN’s performance when all AF information was masked performed well with AP=0.86 and AUC=0.94. We additionally examined MetaRNN’s performance for variants located in genes not seen by the model during training (MetaRNN_UnseenGenes). We identified 10,846 variants in 3971 qualified genes in AAFS for this analysis. The figure shows that the MetaRNN_UnseenGenes showed a similar AUC but lowered AP compared to the MetaRNN model benchmarked using the complete AAFS. This observation demonstrated that our model generalizes well to variants in genes with no available labeled data.

## Discussion

This study proposed two supervised deep learning models to effectively distinguish pathogenic nsSNVs and nfINDELs from benign ones. Compared to other competitors, MetaRNN showed improved overall AUC and specificity across various test data sets, especially those with only rare or de novo TPs and TNs. The improved performance can be attributed to several factors. First, allele frequencies were used as features. Some evidence and observations from our study have shown that population allele frequency can provide valuable information to help separate pathogenic from benign variants [6, 7, 12]. Our training data, which removed only those “easy” benign nsSNVs observed in all populations, seem to be a good trade-off between posing a difficult enough training set for the model to learn useful information from and preserving valuable information from AF features. In future development, different modes of inheritance of diseases and penetrance can be incorporated into model development, making AF information even more useful. Second, information from nsSNVs flanking the target variant helps predict the pathogenicity of the target variant. Our results indicate that incorporating annotations from context nsSNVs, which were previously neglected by other computational tools, can help improve model performance.

Improved score interpretability is another highlight of the models. As clinical laboratories report candidate variants mainly based on the ACMG-AMP guidelines [64], reliable and robust computational approaches can be a cost-effective way of providing supporting evidence for variant interpretation (such as the PM4 and PM5 criteria from the guidelines). By correctly assigning more VUSs into functional groups (pathogenic/benign), more de novo variants or variants with insufficient evidence are likely to be interpreted, leading to an improved diagnostic rate in rare Mendelian disorders.

As illustrated previously, MetaRNN and MetaRNN-indel scores are compatible, which filled another gap by providing a one-stop annotation score for both types of variants. This improvement is expected to be applicable across various settings, such as integrated (nsSNV+nfINDELs) rare-variant burden tests for genotype-phenotype association. Even though NGS-based studies such as whole-exome sequencing studies are designed to detect rare genetic variants, their ability to systematically assess rare genetic variants’ contribution to human diseases and phenotypes still lags behind due to insufficient power. This contributes to both the low AF of the detected variants and relatively low sample sizes compared to genotype-based studies [65]. Using computational prediction scores as weights in burden tests is able to increase the power of such studies [66]. The power increase will be more prominent when nsSNVs and nfINDELs are analyzed in an integrated fashion instead of being analyzed separately.

We provide predictions for all potential nsSNVs (~86 million) in the dbNSFP [27, 28] database for rapid and user-friendly analysis and a GitHub page for stand-alone annotation of nfINDELs (and nsSNVs). The program takes a standard VCF file as input and provides variant pathogenicity scores in a transcript-specific manner as output (supported by ANNOVAR [61]). The average prediction time for a single insertion/deletion is approximately 0.2 s, which can support timely large-scale predictions.

## Conclusions

In this study, we developed two models, namely, MetaRNN and MetaRNN-indel, for the pathogenicity prediction of nsSNVs and nfINDELs. Our models provide improved performance with the following innovations. First, we used flanking region annotations around the target variant to help boost model performance. Second, we focused our predictions on rare variants, which is one of the major gaps in our ability to interpret sequence variants effectively. Third, we provide compatible models on both nsSNVs and nfINDELs to make predictions for these two classes of variants comparable. Last, we provide pre-computed scores for all possible human nsSNVs and a stand-alone program for a fast one-stop annotation of both nsSNVs and nfINDELs. In conclusion, with improved prediction accuracy, score interpretability, and usability, MetaRNN and MetaRNN-indel will provide a more accessible and accurate interpretation of rare VUSs for exome-sequencing-based Mendelian disease studies and integrated (nsSNV+nfINDELs) burden tests for common disease studies.

## Supplementary Information

**Additional file 1: **Includes 11 tables for training and testing data sets used in the study. The names of the tables are: **Table S1.** Training variants for MetaRNN; **Table S2.** Training variants for MetaRNN-indel; **Table S3.** Summary statistics for test datasets used to evaluate MetaRNN; **Table S4.** RNTS validation data set for MetaRNN model evaluation; **Table S5.** RCTS validation data set for MetaRNN model evaluation; **Table S6.** AF-RNTS validation data set for MetaRNN model evaluation; **Table S7.** AAFS validation data set for MetaRNN model evaluation; **Table S8.** TP53 validation data set for MetaRNN model evaluation; **Table S9.** Cancer somatic hotspot validation data set for MetaRNN model evaluation; **Table S10.** ClinVar validation data set for MetaRNN-indel model evaluation; **Table S11.** Search space of hyperparameters for MetaRNN and MetaRNN-indel.

**Additional file 2: **Includes 9 figures of additional results. The names of the figures are: **Figure S1.** Performance (AUC) of different methods benchmarked using the rare nsSNV test set (RNTS, test set 1); **Figure S2.** Performance (AUC) of different methods benchmarked using the de-novo rare ClinVar test set (DN-RCTS, test set 3); **Figure S3.** Performance (precision-recall curve) of different methods benchmarked using the de-novo rare ClinVar test set (DN-RCTS, test set 3); **Figure S4.** Performance (AUC vs. average precision-recall) of different methods benchmarked using the rare ClinVar test set (RCTS, test set 2); **Figure S5.** Performance (AUC) of different methods benchmarked using the all-allele-frequency set (AAFS, test set 4) ; **Figure S6.** Performance (AUC) of different methods benchmarked using DM nsSNVs from HGMD and rare variants from gnomAD (test set 7); **Figure S7.** Performance (AUC) of different methods benchmarked using TP53 test set (TP53TS, test set 5); **Figure S8.** Performance (AUC) of different methods benchmarked using cancer somatic hotspot mutations as TPs and population sequencing mutations from DiscovEHR as TNs (test set 6); **Figure S9.** Pooled analysis of MetaRNN and MetaRNN-indel predictions.

## Data Availability

All training data were obtained from dbNSFP v4.1, available at https://sites.google.com/site/jpopgen/dbNSFP [27, 28]. Validation data were obtained from ClinVar at https://www.ncbi.nlm.nih.gov/clinvar/ [23] and HGMD at http://www.hgmd.cf.ac.uk/ [24]. The public version of HGMD is freely available at https://www.hgmd.cf.ac.uk/ac/index.php. Annotation scores and allele frequency information, including 1000GP [19], ExAC [20], and gnomAD [21], were sourced from dbNSFP v4.1, available at https://sites.google.com/site/jpopgen/dbNSFP [27, 28]. The TP53 validation data set was constructed from the TP53 mutation website (https://p53.fr/index.php) [34]. The cancer somatic variant data set was retrieved from a recent publication, available at https://www.biorxiv.org/content/10.1101/2021.04.22.441037v3 [26]. All pre-computed nsSNV scores are available at http://www.liulab.science/MetaRNN. The stand-alone program and source code to make predictions are available at https://github.com/Chang-Li2019/MetaRNN [29] and https://figshare.com/articles/software/MetaRNN_Differentiating_Rare_Pathogenic_and_Rare_Benign_Missense_SNVs_and_InDels_Using_Deep_Learning/19742503 (10.6084/m9.figshare.19742503.v1) [68].

## Abbreviations

nsSNV: Nonsynonymous single nucleotide variant
nfINDEL: Non-frameshift insertion/deletion
RNN: Recurrent neural network
GRU: Gated recurrent unit
TP: True positive
TN: True negative
VUS: Variant of unknown significance
ROC: Receiver operating characteristic
AUC: Area under the ROC curve
AF: Allele frequency
MAF: Minor allele frequency
DM: Disease-causing mutation
SHAP: SHapley Additive exPlanations
