# Supplementary material for: MetaRNN: differentiating rare pathogenic and rare benign missense SNVs and InDels using deep learning
Source: Genome Med. 2022 Oct 8;14:115. doi: 10.1186/s13073-022-01120-z (PMC9548151; doi:10.1186/s13073-022-01120-z)
Supplement: Supplementary file 2 — Additional file 2: Includes 9 figures of additional results. The names of the figures are: Figure S1. Performance (AUC) of different methods benchmarked using the rare nsSNV test set (RNTS, test set 1); Figure S2. Performance (AUC) of different methods benchmarked using the de-novo rare ClinVar test set (DN-RCTS, test set 3); Figure S3. Performance (precision-recall curve) of different methods benchmarked using the de-novo rare ClinVar test set (DN-RCTS, test set 3); Figure S4. Performance (AUC vs. average precision-recall) of different methods benchmarked using the rare ClinVar test set (RCTS, test set 2); Figure S5. Performance (AUC) of different methods benchmarked using the all-allele-frequency set (AAFS, test set 4) ; Figure S6. Performance (AUC) of different methods benchmarked using DM nsSNVs from HGMD and rare variants from gnomAD (test set 7); Figure S7. Performance (AUC) of different methods benchmarked using TP53 test set (TP53TS, test set 5); Figure S8. Performance (AUC) of different methods benchmarked using cancer somatic hotspot mutations as TPs and population sequencing mutations from DiscovEHR as TNs (test set 6); Figure S9. Pooled analysis of MetaRNN and MetaRNN-indel predictions. [file 13073_2022_1120_MOESM2_ESM.docx]

**MetaRNN: Differentiating Rare Pathogenic and Rare Benign Missense SNVs and InDels Using Deep Learning**

Chang Li, Degui Zhi, Kai Wang, Xiaoming Liu

**Fig. S1. Performance (AUC) of different methods benchmarked using the rare nsSNV test set (RNTS, test set 1).**

**Fig. S2. Performance (AUC) of different methods benchmarked using the de-novo rare ClinVar test set (DN-RCTS, test set 3).**

**Fig. S3. Performance (precision-recall curve) of different methods benchmarked using the de-novo rare ClinVar test set (DN-RCTS, test set 3).**

**Fig. S4. Performance (AUC vs. average precision-recall) of different methods benchmarked using the rare ClinVar test set (RCTS, test set 2).**

**
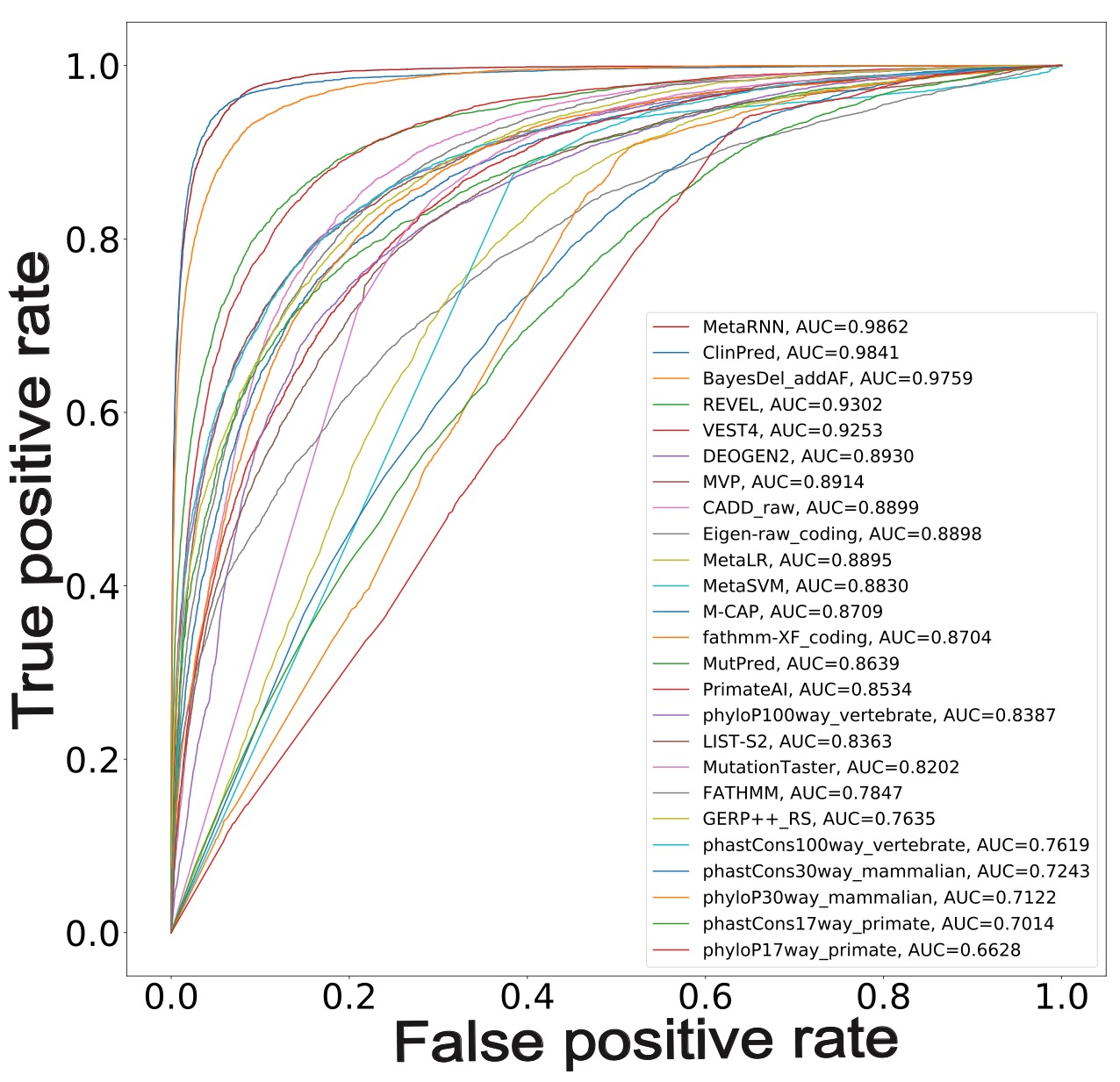
**

**Fig. S5. Performance (AUC) of different methods benchmarked using the all-allele-frequency set (AAFS, test set 4).**


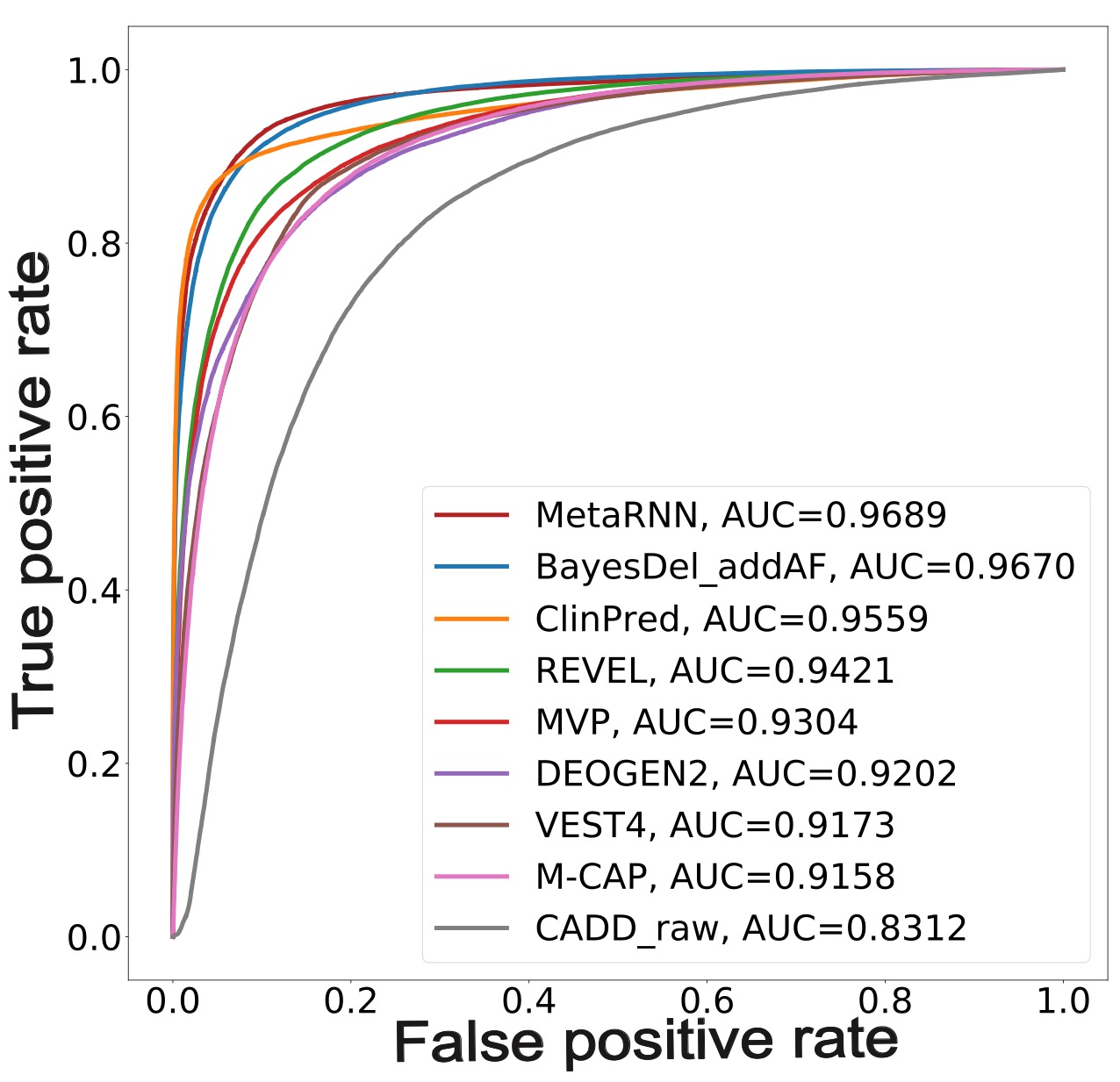


**Fig. S6. Performance (AUC) of different methods benchmarked using DM nsSNVs from HGMD and rare variants from gnomAD (test set 7).**


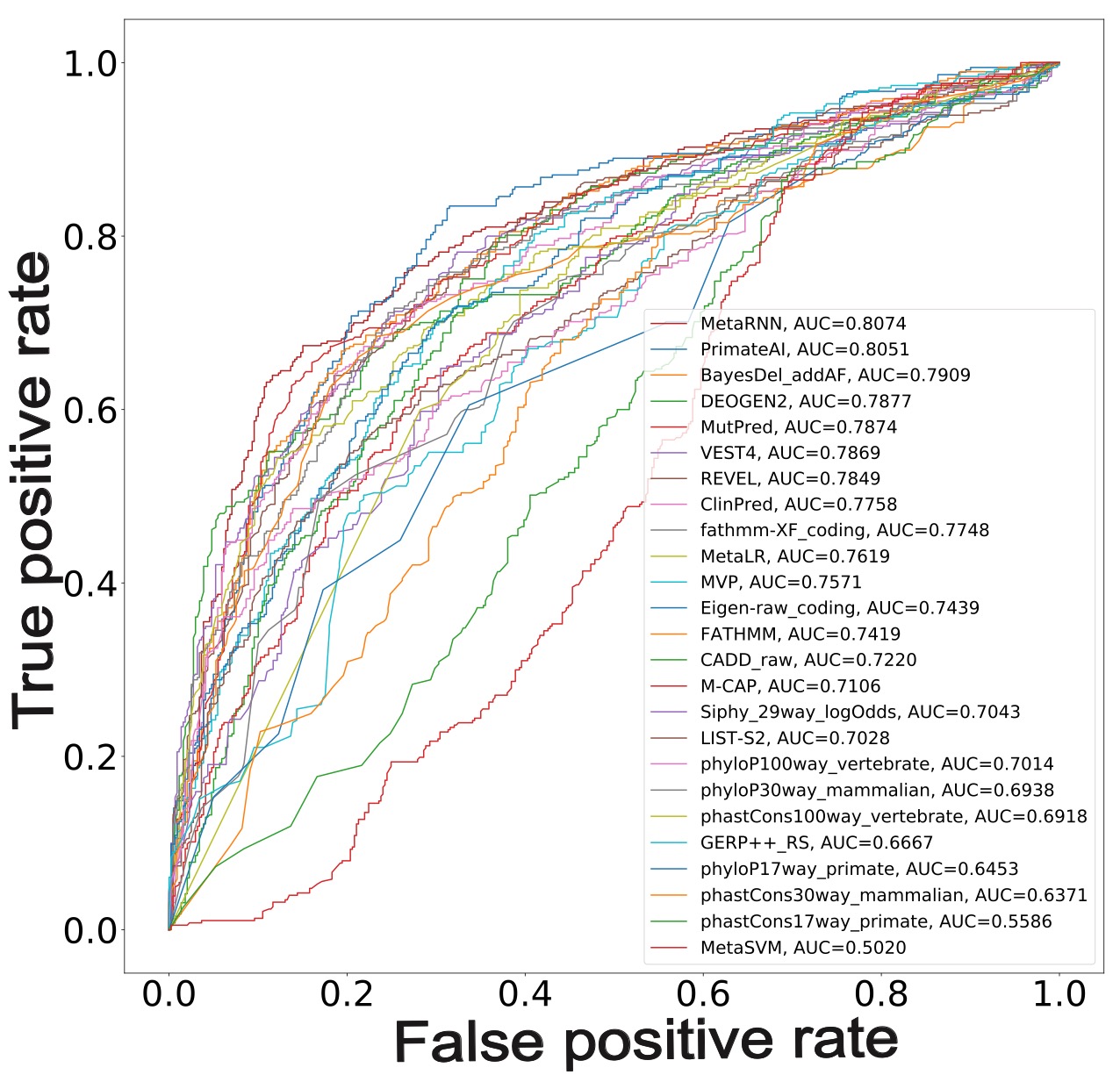


**Fig. S7. Performance (AUC) of different methods benchmarked using TP53 test set (TP53TS, test set 5).**


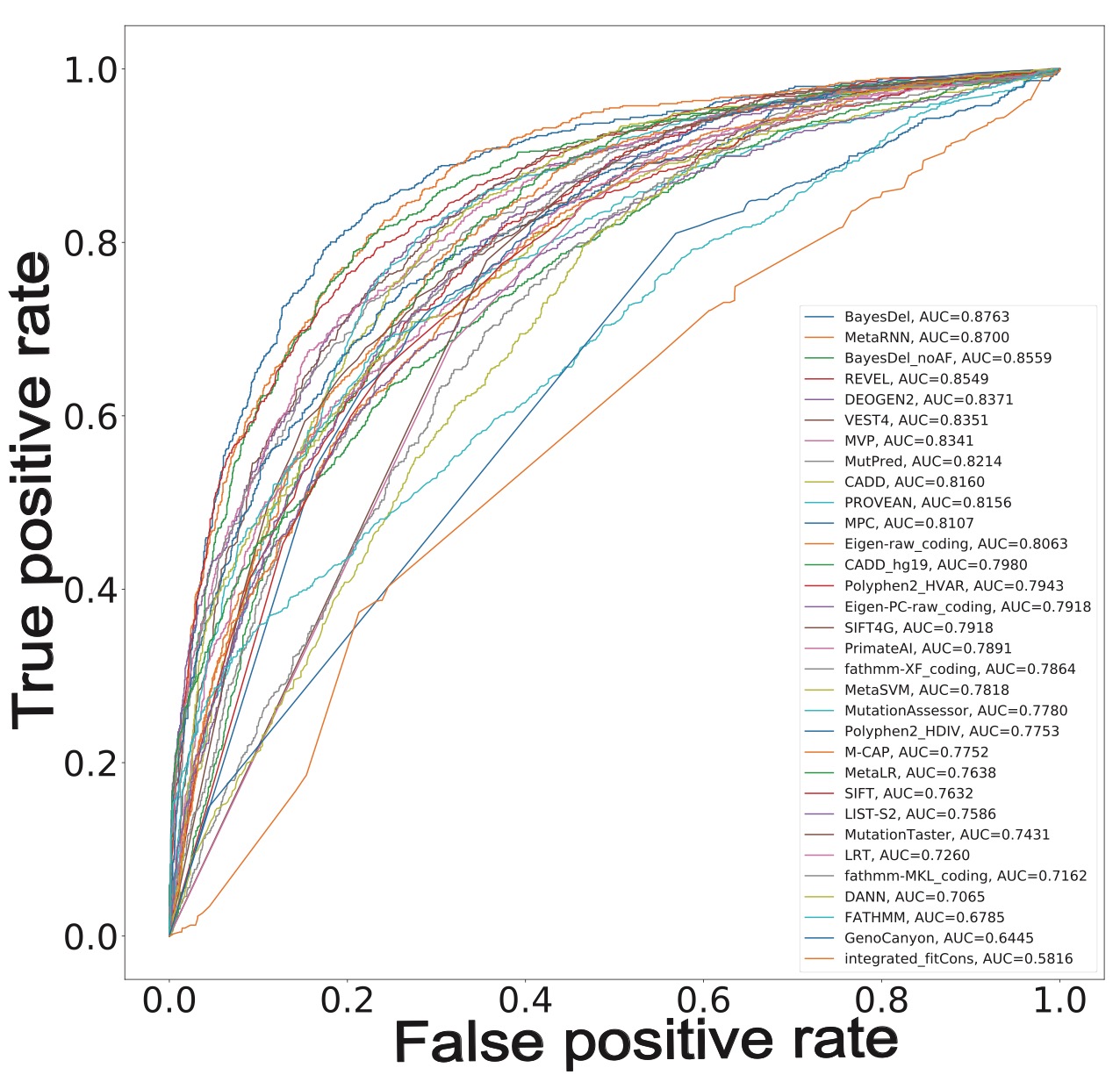


**Fig. S8.** **Performance (AUC) of different methods benchmarked using cancer somatic hotspot mutations as TPs and population sequencing mutations from DiscovEHR as TNs (test set 6).**

**a b**

**Fig. S9. Pooled analysis of MetaRNN and MetaRNN-indel predictions.** From RNTS, 828 random variants were selected to match the number of the ClinVar test set for nfINDELs. a: Distribution of predictions from MetaRNN and MetaRNN-indel by TP and TN. b: AUCs of MetaRNN, MetaRNN_indel and AUC calculated using combined predictions from both models.
